# 

**Published:** 1900-07-14

**Authors:** 


### NOTICE TO CORRESPONDENTS.

**All MS., Letters, Books for Review, and other matters intended for the Editor should be addressed THE EDITOR, "THE HOSPITAL," THE HOSPITAL BUILDING, 28 & 29, SOUTHAMPTON STREET, STRAND, W.C. The Editor cannot undertake to return rejected MS., even when accompanied by stamped directed envelope.**

**Notice to Advertisers and Subscribers.**

All Advertisements, Orders for Copies of THE HOSPITAL, and Business Communications should be addressed to **THE MANAGER (Not to the Editor)**, THE HOSPITAL, The Hospital Building, 28 & 29, Southampton Street, Strand, London, W.C.

The Cost of Subscription, post free, is as follows:—Per week, 2½d.; per quarter, 2s. 8d.; per half-year, 5s. 3d.; per annum, 10s. 6d. Foreign:—Per annum, 15s. 2d., payable, in all cases, in advance.

**NOTICE.—Vol. XXVII. of "The Hospital" (October, 1899, to April, 1900), in handsome cloth binding, is now on sale at the Office of this Journal, price 7s. 6d. Cases for binding the Numbers contained in this Volume 1s. 6d. Index to Vol. XXVII., 2½d., post free. The Monthly Part for July is now ready. Price 6d., by post 8d.**

### The Student of Medicine.

**APPOINTMENTS.**

K. Gimson, M.B., B.C.Cantab., appointed Medical Officer of Health to the Witham Urban District Council. D. L. Hamilton, L.R.C.P., F.R.C.S.Eng., appointed Medical Officer for the Timsbury District of the Clutton Union. E. W. St. Clair Hannah, M.B., Ch.B.Aberd., appointed House Surgeon to the Kent County Ophthalmic Hospital. J. Keating, L.R.C.P., L.R.C.S.-Edin., appointed Certifying Factory Surgeon for the Ballygarvan Dispensary District, co. Cork. T. J. Kelly, Lic. Med. T.C.D., L.R.C.S.I., appointed Certifying Factory Surgeon for the Enniscorthy No. 1 and No. 2 Dispensary District. W. S. Limrick, L.R.C.P., L.R.C.S.,Edin., appointed Medical Officer of Health for the Great Crosby Urban District. J. F. Mannix, L.R.C.P., L.R.C.S.I., appointed Certifying Factory Surgeon for the Caherciveen District, co. Kerry. C. D. Marshall, F.R.C.S., appointed Assistant Surgeon to the Royal London Ophthalmic Hospital (Moorfields Eye Hospital), City Road, E.C. H. Armistead, M.B., C.M.Edin., appointed Medical Officer for the Oswaldtwistle District of the Blackburn Union. H. T. Barron, M.B.Lond., L.R.C.P., M.R.C.S.Eng., appointed Medical Registrar to the Westminster Hospital. J. Chestnutt, L.R.C.P., L.R.C.S.Edin., appointed Certifying Factory Surgeon for the Howden District. R. Cruickshank, M.B., appointed Certifying Factory Surgeon for the civil parishes of Cruden and Slains, county Aberdeen. H. P. Dalley, L.R.C.P., L.R.C.S.Edin., L.F.P.S.Glasg., appointed Medical Officer for the Syston District of the Barrow-upon-Soar Union. R. Emmett, M.R.C.S.Eng., L.R.C.P.Lond., L.S.A.Lond., appointed Medical Officer to the Portsmouth Post Office. J. F. Gillett, M.B., B.C.Camb., appointed Medical Officer for the Second District of the Andover Union. T. D. Nicholson, M.B., C.M.Edin., appointed Certifying Factory Surgeon for the Shap District.

